# Courage, Justice, and Practical Wisdom as Key Virtues in the Era of COVID-19

**DOI:** 10.3389/fpsyg.2021.647912

**Published:** 2021-03-26

**Authors:** Blaine J. Fowers, Lukas F. Novak, Alexander J. Calder, Robert K. Sommer

**Affiliations:** Department of Educational and Psychological Studies, University of Miami, Coral Gables, FL, United States

**Keywords:** COVID-19, flourishing, frailty, practical wisdom, courage, justice

## Abstract

Fowers et al. (2017) recently made a general argument for virtues as the characteristics necessary for individuals to flourish, given inherent human limitations. For example, people can flourish by developing the virtue of friendship as they navigate the inherent (healthy) human dependency on others. This general argument also illuminates a pathway to flourishing during the COVID-19 pandemic, the risks of which have induced powerful fears, exacerbated injustices, and rendered life and death decisions far more common. Contexts of risk and fear call for the virtue of courage. Courage has emerged more powerfully as a central virtue among medical personnel, first responders, and essential workers. Longstanding inequalities have been highlighted during the pandemic, calling for the virtue of justice. When important personal and public health decisions must be made, the central virtue of practical wisdom comes to the fore. Wise decisions and actions incorporate the recognition of relevant moral concerns and aims, as well as responding in fitting and practical ways to the specifics of the situation. Practicing courage, justice, and practical wisdom illuminates a path to flourishing, even in a pandemic.

## Introduction

The year 2020 has been one of the most tumultuous, uncertain, and defining in living memory, with the COVID-19 pandemic and global political unrest vying with World War II, the Great Depression, and the Flu of 1918 as among the greatest challenges to physical, mental, and social health in modern times. As the virus rages across the globe, hospitals and medical personnel are being overwhelmed: as of February, 2021, there have been over 100,000,000 confirmed COVID-19 cases and 2,000,000 COVID-19 related deaths worldwide; in the United States alone, there have been over 27,000,000 confirmed COVID-19 cases and over 400,000 COVID-19 related deaths (WHO, 2021). It is heart-breaking that many of those deaths occur without the comforting physical presence of loved ones and communal mourning has been extremely limited. The painstaking development of new treatment regimens and newly released vaccines are the sole rays of hope in many countries, including the United States (Johns Hopkins University Coronavirus Resource Center, 2020).

The coronavirus’ impact, however, extends much further than its biological course or mortality rate can express. The pandemic has cost tens of millions of people their loved ones, their jobs, their businesses, and their food security. Although a very helpful public safety tool, social distancing measures have increased social isolation and uncertainty amid the unprecedented rates of psychological distress during the pandemic (Fowers and Wan, 2020). Accordingly, mental health has been adversely affected in many populations (e.g., Munasinghe et al., 2020; Gallagher et al., 2021; Yıldırım et al., 2021), but various indices of positive outlook, hope, and resilience have been found to buffer that stress.

COVID’s widespread impact is unlike anything the modern world has seen. When one couples the pandemic with the global social unrest related to police violence against people of color and the vitriolic partisanship and nationalist politics of our times, it can seem as though the human world is coming apart at the seams. We begin this article by identifying three thematic difficulties exacerbated by the pandemic: risk, injustice, and complexity. We then discuss how the virtues of courage, justice, and practical wisdom can help us to handle these difficulties and illuminate a pathway to flourishing, even during a pandemic. We do not claim that the difficulties of risk, injustice, and complexity are the only difficulties exacerbated by the pandemic, nor do we claim that the three virtues of courage, justice, and practical wisdom are the only useful virtues in addressing difficulties exacerbated by the pandemic. We highlight these three difficulties and these three virtues for their salience in the pandemic, and because they provide a compelling account of the relevance of a neo-Aristotelian virtue perspective for practical psychological life. We see this as worthwhile especially as this neo-Aristotelian perspective parallels Positive Psychology 2.0 (PP2.0).

## Risk

There are three intensifying features of the risks we face amidst this pandemic and social unrest. First, we are reckoning with threats that are largely invisible, yet threaten our lives and livelihoods. Second, although both the virus and racialized violence are continually roiling through societies, either one can burst out suddenly and unexpectedly. Widespread uncertainty and fear result partly from the slow, painstaking acquisition of knowledge and the continued search for effective treatments and shortages of vaccines and testing supplies. These multi-faceted risks can be summarized as both uncertain certainties (e.g., the possibility of death, the higher likelihood of the virus adversely affecting older populations and those with pre-existing medical conditions) and certain uncertainties (e.g., the exact risk each individual has, precisely what type of exposure is needed to contract the virus). Finally, to make matters worse, most people are dealing with these difficulties with far greater social isolation than in ordinary times. Social connections are primary contributors to well-being as well as stress moderators. We are having to dig deep in our individual and collective resources to cope with and manage these challenges. We will argue that courage is the virtue that is central to addressing risk well.

## Injustice

The pandemic has also shined a harsh light on longstanding inequity and injustice in the degree of societal justice involving the distribution of harms and benefits^1^. A category of workers has acquired newfound recognition, although they have always been with us: “essential workers.” Those officially referred to in the United States as “essential critical infrastructure workers” (healthcare, pharmaceutical, and food supply workers) were informed by the United States Department of Homeland Security in 2020 of their “special responsibility to maintain their normal work schedule” (Krebs, 2020). Unlike the large number of workers who could transition to remote work to prioritize their health and safety, essential workers were mandated by the executive branch of the United States government to put the health and safety of others ahead of their own. Although it is heartening to have these workers’ value recognized explicitly, their jobs remain low-paying and disproportionately populated by people of color (Long et al., 2020). Despite the designation of “essential,” a recent examination pointed out that although we often celebrate people working in medicine, “we may think of course of the nurses and doctors treating patients; but we ought to think too of the people scrubbing and disinfecting the walls and floors” (Kinder, 2020). One worker expressed this lack of recognition by saying, “people are not looking at people like us on the lower end of the spectrum. We’re not even getting respect.” Others state their yearning for appreciation and recognition, a desire for “a thank you. ‘I am glad you are here, thank you for coming to work.’ Hazard pay. Anything.” (Kinder, 2020). In addition, the very act of deeming some workers “essential” has the unintended effect of devaluing the work of others who have lost their livelihoods (e.g., hospitality, entertainment, and arts workers) and whose work has not been recognized as sufficiently important to the good of our communities to merit the risk it entails.

In addition, everyone understands that medical professionals, first responders, and military personnel take on occupational risks beyond what other workers accept. These workers understand they must take outsized risks during crises and that they will often be the first ones called upon to take those risks. For these workers, the pandemic intensified pre-pandemic demands, and they were trained and equipped to engage with issues of life and death. In contrast, supermarket employees or postal service workers have not historically needed to consider such stress or intensity when accepting or conducting their jobs. Therefore, there are many millions who have been required to perform “front line work” who neither signed up for nor are compensated adequately for these risks. This seems to be an inequitable distribution of very consequential risk.

It is also necessary to recognize the historical injustice regarding access to health care in the United States, and the disproportionate toll of illness and death from COVID-19 suffered by people of color (Centers for Disease Control and Prevention, 2020) with, for example, Blacks comprising just under 13% of the population (United States Census Bureau, 2020) and making up 17% of COVID-19 deaths. This disproportionate suffering, death, and loss have highlighted the history of less adequate medical care and support for health available to these populations. For these reasons, we will argue that people with the virtue of justice are needed to work toward a fairer sharing of burdens and benefits.

## Complexity

The complexity of the pandemic is another of its vexing features. Governments across the globe have confronted difficult issues about how to communicate with the public about risk, risk-management, changing information due to slowly increasing knowledge, and changing information about the likely future. Governments have also struggled with where to allocate resources to combat the virus and its effects. Many of these difficulties involve grappling with what seemed like contradictory or at least conflicting goals such as how to keep a country and its economy afloat, while also keeping its citizens safe and healthy. Some governments emphasized safety over the economy, some focused more on the economy than on safety, and some attempted to balance the two.

There are also extremely complex, consequential decisions forced on medical personnel as health care systems become increasingly overwhelmed. Difficult decisions are necessary in triaging and treatment planning given the limits on equipment and personnel. How does one estimate the likelihood of successful treatment with such an unpredictable illness? It is overwhelming to be in a position that seems to require determining which life is more valuable or worth saving than another.

This complexity is apparent in business and education as well. Many small businesses face the serious threat of going out of business permanently, with potentially devastating financial, social, and emotional consequences. Choosing or being compelled to prioritize the safety of the community through closing, or even severely reducing the number of people they can serve, has led to widespread job and business losses. Tough decisions have also been necessitated in education. There is little doubt that students and teachers alike prefer to be in the classroom environment over tele-classrooms, and that being in the physical classroom has long-lasting academic and social benefits that virtual education does not. Yet, government officials and school administrators must decide how to prioritize the health and well-being of students, staff, and teachers, the ability of parents to work, the educational benefits of in-person schooling, and the many other services schools provide.

Many personal decisions during the pandemic are challenging. A common thread in decision-making throughout COVID-19 has been attempting to balance what is best for an individual or “in-group” with what is best for others or the collective. For many, including medical professionals, first responders, and essential workers, the combination of risks to their own safety, their patient’s safety, and to their friends and families’ safety make for difficult decisions. Given the extremity of needs, one could choose to work over-time, which may appear noble and other-focused, but also may result in work-undermining fatigue or becoming ill and may put their colleagues at increased risk as well.

Another set of complex decisions is necessary for individuals needing non-COVID medical procedures. Given the danger associated with going to the hospital during the pandemic, and the limited resources and under-staffing many institutions face, many patients have chosen to delay treatments even for critical care such as heart attacks and strokes. In addition, many surgeries or appointments have been deemed by medical centers as “elective,” leading to cancelation or rescheduling. Although this rescheduling will not have dire consequences for many, there are others with serious, even life-threatening illnesses other than COVID (e.g., cancer treatment). We do not think there are any simple or universal answers for any of these complexities, but we do think they can be handled more or less wisely, so we will discuss how the virtue of practical wisdom can help.

## Summary

The risks, systemic unfairness, and complexities of the pandemic have been extremely difficult for individuals and societies to manage, and there are many disagreements about how to best respond to these intensified problems. Clearly, some leaders and individuals have managed the difficulties better than others and understanding the capacities that make good leadership (and followership) possible can be beneficial today and in the future.

The key resources we discuss in this article are individual virtues and the collective encouragement and support for virtuous action. It is important to make explicit reference to the communal aspects of virtues, so that we are not misunderstood to see virtues as entirely internal to individuals. We must keep in mind that people are taught virtues by others, that communities and societies can value and promote virtues or devalue them, and that individuals are strongly influenced by others in their actions, virtuous or otherwise. Our view is that human action is always deeply and inextricably entwined with others, which, of course, includes virtuous action (Fowers, 2015, 2017; Fowers et al., 2017).

In this article, we will focus on the virtues of courage and justice, and the meta-virtue of practical wisdom. Situations of threat or risk call for the virtue of courage, balancing burdens and benefits in a consistently fair manner requires the virtue of justice, and when complex, multidimensional, and consequential decisions must be made, practical wisdom comes to the fore. We will argue that these virtues are necessary precisely because humans are frail creatures who can easily go wrong when at risk, balancing burdens and benefits, or in need of making wise decisions. A more general case for virtues as the very characteristics that make it possible for one to act well in circumstances that reveal our human limitations was made by Fowers et al. (2017), who discussed friendship as the excellence associated with dependency, compassion as the virtue involved in suffering, and reverence and humility as the virtues involved in human limits. Because human limitations are always evident and life is challenging even in ordinary times, the virtues become the most promising pathway to flourishing as a human being, even in pandemic times.

## What Is Virtue?

Many scholars use the term *virtue* colloquially, which may be sufficient for some purposes, but because this paper focuses specifically on virtues, it is incumbent on us to provide a clear conceptualization of virtue. Broadie (1991) described virtues simply and succinctly by saying that, “an excellence or virtue, as Plato and Aristotle understand that concept, is nothing but a characteristic which makes the difference between functioning and functioning well” (p. 37).

We take a neo-Aristotelian approach to virtues that portrays them with multiple features: Virtues (1) are acquired traits that (2) vary in strength across individuals, (3) are responsive to social roles, (4) are sensitive to the specifics of the situation, (5) facilitate the pursuit of valued aims, (6) make it possible to live well, (7) show up in behavior, (8) are based on knowledge, (9) are fully and properly motivated, and (10) are guided by practical wisdom (Fowers et al., 2021). Virtuous action means that one knowingly chooses to act in ways that conduce to worthwhile goals, given the specifics of the situation, and with the kind of harmonious motivation and emotion that arises from having a settled disposition to do so. In addition, Aristotle (1999) suggested that virtues are the most fitting response to a given situation, being flanked by a vice of deficiency and a vice of excess. We discuss each of these features in what follows. We exemplify these features with the virtue of courage because it is so salient during this pandemic.

Of course, we recognize that our portrayal of the relationship between virtue ethics and flourishing is one among many, and we are open to critique and reformulation based on other perspectives. Other scholars have conceptualized this relationship differently and emphasized other domains, such as psychotherapy (Wong, 2017; Jankowski et al., 2020) and organizational behavior (Newstead et al., 2018). Perhaps most similar to the neo-Aristotelian approach we take is within PP 2.0 which sees virtue as a critical aspect of improving the lives of people and the functioning of society as a whole (Wong, 2011).

### Acquired, Scalar Traits

We see virtues as traits that are acquired through practice and guidance, and those traits vary across individuals. We suggest they are traits because Aristotle (1999) focused on virtues as “settled dispositions” that are stable and reliable. If one is to act courageously, especially when that action is called for suddenly and unexpectedly, as often occurs, one must be prepared to be courageous by having already acquired the trait. Importantly, we suggest that virtues are scalar (vary in strength across individuals) as opposed to the view that one either has a complete virtue or does not (Cokelet and Fowers, 2019).

### Sensitive to Roles and Situations

Although we see virtues as traits, this does not mean that anyone would exhibit courage constantly and in exactly the same ways across roles and situations. One of the aspects of virtues that the pandemic has highlighted is that roles are extremely important. This shows up with courage in that medical personnel, first responders, and a wide range of essential workers have been called upon by society to put their health at greater risk (act more courageously) than people in many other roles. These work roles place special demands on the individuals who fulfill them. For example, medical professionals risk their own health and safety (and that of their loved ones) each time they work a shift. In addition, situational variation means that courage will be evident in many different ways in a single individual’s experience. For example, food delivery workers must risk exposure to the virus to bring necessary food to others at work, but when they are not working, taking a similar level of risk would not be advisable. If one is caring for someone who is ill with the virus, that caring will require greater risk taking than the periods before or after the caregiving. This variation means that there is no one right way to be courageous, and that the demands of courage vary widely, between and within individuals and across situations and roles.

### Valued Aims and Living Well

The reason that virtue theorists believe that roles and situations provide important guidance for virtue expression is that roles and situations often afford the pursuit of specific valued aims, such as health, public safety, and dignity. The reason it is sensible for medical personnel to put their health at risk is that they are working to maintain or re-establish the health of their patients. The reason that public demonstrations led by social justice advocates are worth the risk to public health is the aim of greater social justice. In other words, the justification for taking risks is that there is something important at stake. From a neo-Aristotelian perspective, risk-taking is not courageous when it serves lesser ends such as thrill-seeking or showing off.

We see discussions of virtues that do not recognize the linkage of virtue and the pursuit of a good life as incomplete. In our view, virtues are the characteristics that enable people to reliably and successfully pursue worthwhile ends, such as public health. From our neo-Aristotelian perspective, the best way to live is to experience a felicitous combination of worthwhile aims, such as belonging, societal justice, health, and social harmony. The focus on these elements of a good life is a key premise of Aristotle’s ethics and it differentiates his view from more common contemporary ethics, which tends to focus on doing the right thing based on ethical principles or the consequences of actions. That is, Aristotle saw the aim of ethics as promoting the best possible life for oneself and one’s communal world, and that includes multiple goods rather than a singular good.

### Behavior

Virtues must be evident in action to qualify as virtues. For the virtue of courage, it is obvious that brave thoughts or feelings are insufficient without acting in ways that involve risk taking. Of course, virtuous courage is not appropriate to every situation, but when a circumstance calls for courageous action, a person with the virtue of courage will take the appropriate risks to seek or maintain worthwhile aims.

### Knowledge

Just as brave thoughts are insufficient, so is behavior that is not guided by knowledge about courage and the knowing intent to act courageously. Knowledge is important because when a person incidentally or accidentally takes risks, this cannot count as courage. The virtue of courage requires that one knowingly takes risks that are justified by acknowledged and valued aims.

### Emotion and Motivation

Our view is that virtues require concordant emotion and motivation. Courage is the virtue that enables people to confront fear-inducing situations excellently. This is not to say that a courageous person is completely fearless. Many circumstances evoke fear, especially in the current pandemic: caring for an ailing loved one, being deemed an essential worker, being a member of a marginalized group during a time of increased violence, or working on the front lines of the medical, public policy, or policing professions. Indeed, one way to go wrong in the domain of risk-taking is failing to recognize real risks that properly evoke fear. Many situations engender fear in reasonable people. Being able to appropriately respond to fear-inducing situations does not mean that fear itself is problematic or should be eliminated. Rather, fear is a basic and powerful emotion that helps us recognize and respond to dangers.

In confronting fears, we must take risks to our physical or mental health, our social standing, and so forth. Risk taking only counts as a virtue when one willingly takes the risk and is fully motivated to do so. This concordance of behavioral and motivational states is discussed in terms of duty and desire. Acting virtuously means wanting to act in ways that seem best, without significant conflict between desire and duty. When one acts in a good way, but does not want to, this is a conflict between desire and duty known as continence (Aristotle, 1999; Fowers, 2008). Continent action is clearly worthwhile, but it is both easier and better when one wholeheartedly wants to do what is for the best. Courage is a virtue precisely because one acts resolutely despite one’s justified fears. That is, courage is not the lack of fear, but its mastery.

### Practical Wisdom

According to Aristotle (1999), virtues such as courage are guided by practical wisdom (*phronesis*), which is what makes practical wisdom a meta-virtue. One of the main functions of practical wisdom is to clarify what courage, for example, consists in, by taking into account both the dangers and opportunities of the given situation and the worthwhile aims that can be pursued. This shows up clearly in Aristotle’s formulation of the structure of virtues, being flanked by vices of deficiency and excess. Courage is an excellent example of this structure because courage is called for in situations of risk and danger. Enacting the virtue of courage means taking the appropriate amount of risk given both the degree of danger and the ends (e.g., health, safety) that are at stake. When one shrinks from taking risks that are justified by the ends, this is a deficiency of risk-taking, often termed cowardice. Avoiding necessary risks may represent an excessive sensitivity to fear. Suppose a doctor is excessively sensitive to fear, and that doctor freezes instead of providing appropriate care to a very sick patient. Inaction would be a significant failing because inaction would not promote the good of the patient’s health. When one takes risks that are too great in view of the ends at stake, this is an excess of risk-taking, also called recklessness. For example, suppose a doctor were completely insensitive to fear and rushed into treating the patient without following established safety protocols. This doctor would fail to recognize the danger of the situation and therefore take excessive risks to his or her health. Practical wisdom is the capacity to properly recognize the appropriate degree of risk-taking. That is, practical wisdom helps us to recognize what courage amounts to, given both the specifics of the situation and the ends at play.

The COVID pandemic has clarified how situations and roles affect our judgments about the risks that are worth taking. Risking one’s health, indeed one’s life, has become far more common in this time, and those risks are regularly undertaken by medical workers, first responders, and people who produce and transport vital goods for others’ health (e.g., food, medicine). This risk-taking is courageous because the ends are so valuable that sizable risks are justified. In contrast, taking serious risks for the sake of entertainment or to obtain trivial items is seen by public safety experts as reckless because those ends do not warrant the danger incurred. Put another way, the dangerous risks undertaken by a nurse count as courage, whereas similar risks would be excessive for an ordinary person going to a neighborhood party. In addition, the nurse has extensive training, access to the necessary safety equipment (usually), and a team of professionals, all of which increases safety. Nurses generally take the risks they do knowingly, intentionally, with concordant motivation and emotion, and on a foundation of training.

In this context, it is easy to see why Aristotle did not propose that there is a simple and singular way to enact the virtues because variations in situational specifics and role requirements are potentially endless. Rather, Aristotle proposed a meta-virtue, practical wisdom, which entails attending to the morally salient details of a situation, planning actions, and integrating reason and emotions. We turn now to discussing the three salient virtues we have selected for emphasis in this paper: courage, justice, and practical wisdom.

## Courage as a Pathway to Flourishing Through Frailty

Human beings are frail creatures because we can be harmed physically, psychologically, socially, or materially. It is important that we reckon with these dangers with open eyes so that we can successfully manage the risks to our safety and well-being. The emotion of fear is a powerful part of human harm avoidance that appears to have analogs in virtually all vertebrates (LeDoux, 2012). The perception that a risk is significant can lead to autonomic nervous system arousal, and freeze, flight, or fight behavioral responses. During the pandemic, perceived risk is a significant predictor of depression (Gallagher et al., 2021; Yıldırım et al., 2021). Therefore, humans appear to be naturally endowed with fear, which evolved because it helped our ancestors to avoid harms that impeded reproduction. That is, fear evolved because it helps us deal with our frailty.

We agree with Aristotle (1999) that humans have choices in how we respond to our circumstances, and situations of risk are generally no exception. Of course, risk and fear can be so overwhelming that no choice is possible, but this extremity is fortunately rare in ordinary life. The neo-Aristotelian aim is to habituate good choices and actions in response to situations that arise, and these responses are the virtues. In cases of risk and fear, courage is required. Recall that courage is understood as the appropriate degree of risk-taking, and that one can go wrong by shrinking from taking risks to pursue a valued aim (deficiency of cowardice) or by plunging into unnecessary or unwarranted risks given the ends at stake (excess of recklessness).

Our neo-Aristotelian view is that flourishing as a human being is a matter of regularly participating in a wide range of human goods, such as health, safety, pleasure, friendship, and so forth. These goods are frequently threatened in various ways. The pandemic is an excellent example of a threat that puts virtually all human goods at risk. COVID-19 is an obvious risk to health and life itself, but the recommendations to socially distance, wear masks, and avoid congregating also threaten our enjoyment of life, our relationships with others, and our social cohesion. The lack of regular, in-person contact with people with differing backgrounds may also threaten our ability to promote social justice.

As Aristotle (1999) pointed out, gods do not have to worry about threats to their well-being, but humans do. Because participating in human goods is the way to flourish, and those goods can be threatened, the capacity to respond courageously to those risks is necessary for a good human life. The courage that has been so prominently and consistently displayed by so many people during this pandemic has been inspiring. Conversely, the recklessness of many people has deepened and prolonged the pandemic, contributing to unnecessary suffering and death. Because it is public health that is at stake, we endorse Aristotle’s (1999) exhortation to “feel [fear and confidence] at the right times, with reference to the right objects, toward the right people, with the right motive, and in the right way, …and this is characteristic of virtue” (1106 b 17–23). Of course, having good judgment about risk-taking is no simple matter, and Aristotle presented practical wisdom as the capacity to make good decisions and take appropriate action, which we discuss more fully below.

## Justice as a Pathway to Flourishing Through Frailty

Courage has been widely recognized during the COVID-19 pandemic, but it is also clear that the risks and benefits of the pandemic have not been equitably distributed. Aristotle (1999) famously called humans “political animals,” and one way to understand politics is in justice terms: the process of distributing burdens and benefits. Justice can refer to both a societal arrangement of those distributions and as a virtue. The virtue of justice refers to being a person who is a “performer of just actions” (Aristotle, 1999, 1129a 8). We focus on justice as a virtue in this section.

There are many ways to parse the concept of justice, but in this discussion of the pandemic, we focus on a fair distribution of benefits and burdens. Thus, the virtue of justice is enacted when an individual actively, knowingly, and with proper motivation contributes to an equitable distribution burdens and benefits. This virtue is flanked by a vice of excess, as when people take more than their share of benefits (e.g., vaccines, food, supplies) and less than their share of burdens (e.g., risks, cost). The vice of excess can emerge due to a disproportionate focus on benefiting oneself (egocentricity) or on benefiting one’s group (ethnocentricity). Both biases are common and inimical to well-being (Fowers, 2015).

We see the vice of deficiency arising when one forgoes appropriate benefits and accepts excessive burdens. Aristotle (1999) did not believe that this vice of deficiency existed because he did not believe that one could treat oneself unfairly. Contemporary scholarship on the internalization of oppression (e.g., Gale et al., 2020) has clarified that people who are exploited or stigmatized frequently come to believe that they deserve less and become used to claiming less than their fair share. That is, people can be taught that they deserve less than their fair share by oppression, stigmatization, and marginalization, and this becomes internalized and ascribed to oneself or one’s group rather than to the oppression itself.

In a crisis such as this pandemic, it is common for many people to reflexively take more than their share (e.g., toilet paper hoarding) or to pursue unfair advantage (e.g., vaccinations for the privileged over the vulnerable). Seeking safety for oneself and one’s loved ones is a natural human tendency, but it is also one that can exacerbate societal injustice. Cultivating the virtue of justice prepares one to respond in more equitable ways because injustices are more visible to a just person and that person is also strongly motivated to pursue a fair distribution of burdens and benefits.

There is evidence that just society relations are positively related to individual well-being (Di Martino and Prilleltensky, 2020). Wilkinson and Pickett (2020) draw on extensive data to argue that equality in the distribution of societal resources is positively correlated with many indices of well-being. There is some dispute about this conclusion along with some evidence of no relation between inequality and well-being Ngamaba et al., 2018). Another argument links cooperation, a ubiquitous human inclination (West et al., 2007) with fairness because cooperation collapses without fairness. Cooperation is necessary for humans to flourish, suggesting that fair actions by cooperators is also required (Fowers, 2015).

Concerns about societal justice are likely at least as old as the human species, and there has been an incredible variety of social arrangements and philosophies that address questions of justice (MacIntyre, 1988; Henrich and Henrich, 2007). We offer no definitive answer to those questions here. We do want to emphasize one point, however. Although rules, laws, institutions and social practices are necessary, they are insufficient. This insufficiency is manifest in the racialized violence against people of color by police, the very people entrusted to enforce the laws we have against unnecessary violence as well as the provision of our constitutions that insist on equal treatment before the law. We need just laws and practices, but we also require people who have the virtue of justice to uphold those laws and practices faithfully. Cultivating and practicing the virtue of justice is the way to be a just person in the most consistent and broad way. For these reasons, we claim that some defensible form of the virtue of justice in individuals and in groups is a necessary element of a flourishing life.

## Practical Wisdom

Aristotle (1999) enumerated various virtues, each pertaining to different dimensions of human frailty and the vicissitudes of fortune. These virtues encompass much of what it means to live well. But enacting virtues requires good judgment about when and how they are appropriate to concrete situations and how they can contribute to what is good. This capacity for good judgment is a kind of meta-virtue that guides a person in organizing and integrating the other virtues. Aristotle called this capacity *phronesis*, which has been translated as practical wisdom.

Of course, there is a good deal of debate about what practical wisdom is and is not, with many views among philosophers and social scientists (Grossmann et al., 2020; Kristjánsson et al., 2021). Some see it as a “skill” (Stichter, 2018), others as a kind of intuitive artistry (Dunne, 1993), still others as just one virtue among others (Peterson and Seligman, 2004). We adopt what we see as the most comprehensive contemporary neo-Aristotelian account of practical wisdom here, which was provided by Darnell et al. (2019). Those authors, along with Kristjánsson et al. (2021) provide extensive discussions of what makes this account preferable to others. These two papers make three key points. First, they have designed their model to be consistent with Aristotle’s philosophy as the primary source for most modern discussions of practical wisdom. Second, they have thoroughly embedded Aristotle’s conviction that practical wisdom is, above all, the capacity to fashion a good and moral life. Finally, they have integrated the best contemporary research on moral emotions, moral identity, moral reasoning, and moral decision-making.

Practical wisdom is the capacity to integrate the many virtues into a coherent and worthwhile life. Without practical wisdom, an individual could not live well consistently, because the complexity and contingencies of life would be overwhelming. To take a concrete example that is directly pertinent to the pandemic, first-responders often accept risk in their work to serve those who suffer from the effects of the virus. Accepting this risk requires the virtue of courage. However, extenuating circumstances can complicate the situational reasoning of a first-responder. For a first responder strained by repeated exposure to the dead and dying, it may be most appropriate to sometimes forgo the most demanding aspects of their work, and instead opt for rest. Discerning how to act courageously, and what risks to take in a situation requires an attention to particularity, and the capacity for this sort of discernment is one dimension of practical wisdom.

The pandemic has made the importance of good judgment glaringly obvious for people ranging from government officials to medical personnel to ordinary citizens. Practical wisdom is important even in ordinary circumstances, but everyone has had to make life and death decisions during this period, whether that involves public policy, medical treatment, essential services, or just obtaining groceries and other necessities.

Before we discuss practical wisdom in more detail, it is important to make two general points. First, similar to the virtues, practical wisdom is a capacity that varies between and within persons. No one has perfect practical wisdom, and it is unusual for people to entirely lack this capacity. Individuals who generally make practically wise decisions also make mistakes, and fatigue can lead to lapses in judgment. The best that imperfect people can do is to make practically wise decisions as often as possible and to develop this capacity as fully as possible. Second, Aristotle (1999) differentiated practical wisdom from *sophia* or theoretical wisdom. Theoretical wisdom is concerned with universal and unchanging matters, whereas practical wisdom relates to everyday decisions in practical life, where facts and situations vary continuously.

Darnell et al. (2019) discussed practical wisdom in contemporary terms. Their general description of practical wisdom is very apt:

*Phronesis* is meant to crown, as it were, virtuous habits with a cluster of intellectual abilities and experience that are both necessary and sufficient for ensuring that these habits will not go awry, will be reliable both over time and across different situations, and will be put into practice in a way that is reflective and motivationally robust (p. 16).

This description clarifies that practical wisdom is the overarching capacity to manage virtuous actions in a variety of situations and organize one’s actions into a coherent and well-motivated approach to life.

## Four Components of Practical Wisdom

Darnell et al. (2019) also outlined four major functions that practical wisdom fulfills. This includes recognizing the salient features of the concrete situation, integrating multiple relevant factors into decisions and actions, an overall understanding of what is worthwhile, and guiding emotional responses with reason.

### Moral Perception

The first component of practical wisdom is the ability to perceive what is most important about a given situation so that the relevant virtue or virtues can be activated. We discussed the example of risk or threat toward worthwhile ends as the key feature of a situation that calls for courage. Similarly, the need to balance the benefits and burdens that accrue in a situation calls for justice.

### Integration

The second component is the ability to integrate multiple moral considerations in decisions and actions. Many situations are complex, calling for more than one virtue, and practical wisdom is the ability to integrate and organize those virtues to guide the best actions. This integration is especially salient when there is some degree of conflict between the virtues or one virtue needs to be prioritized over another one. For example, both honesty and compassion are relevant when medical personnel communicate with patients about their conditions and prognoses, and wisdom operates as the appropriate balancing of the two virtues.

### Blueprint Function

The third component is a firm understanding of what is good, which can be thought of as having a blueprint for a good life. This component is often implicit in people’s actions, but, as Aristotle noted, we pursue what we think are worthwhile aims in all our actions. This is usually referenced in terms of goals, but goals are simply concrete versions of what one sees as good. The practically wise person’s understanding of what is good attunes them to certain features of a situation and not others; the benchmark of salience. The practically wise person uses their knowledge of what is good to weigh various concerns against each other, and they invoke that view of the good in translating their integration into practice. Because practical wisdom is so inextricably bound up in the understanding of what is good, it seems obvious that without this component, practical wisdom is rendered empty, because without a view of what is good, there is no basis for *salience* in interpretation, no means for comparison in integration, and no standard by which to evaluate various translations into action.

To illustrate the paramount importance of blueprinting with an example, consider the first responder without this capacity. They would not be sensitive to the importance of lives gained or lost; they might approach their work in an overly technical way; or they might be inured to the importance of their own health and well-being.

### Reason Infused Emotions

Finally, moral emotions such as empathy, awe, guilt, and anger regarding injustice are important parts of every person’s moral life. Practical wisdom makes it possible to infuse these emotions with reason, which allows people to reflect and guide their emotions toward moral actions that are fitting for the situation and consistent with the actor’s overall understanding of what is worthwhile. The practically wise person’s emotions (and actions) must be responsive to reason, where reason tracks worthwhile ends. Without this correspondence, an individual is liable to act impulsively or with excessive or deficient emotions in difficult situations, and thereby fail to pursue the best course of action. For example, a person with a relative with chest pains might feel compassion toward their loved one’s fear of going to the hospital, given the concomitant risks. This compassion could coexist with the knowledge that the relative needs medical attention. If this individual’s emotions and actions are not attuned to reason, they might avoid bringing their relative to the hospital, even though seeking medical care would be the best course of action. This example illustrates that unless emotions (and actions) are responsive to reason, an individual cannot act virtuously. Therefore, emotions (and actions) that are responsive to reason are a necessary component of practical wisdom.

Once the practically wise person has detected the salient features of a situation and integrated those features into a coherent and actionable narrative, they must act. This final step of translation, is eminently specific, hinging as it does on many features of a given situation. The capacity to elegantly and appropriately act in a virtuous way based on an understanding of the contingencies of the current situation and the goods that can be pursued in that situation characterizes a practically wise person.

### Practical Wisdom and the Virtues

Practical wisdom is thought of as a meta-virtue because it is required for the exercise of any particular virtue. There are many virtues, and although the precise taxonomy is contested, their multiplicity is not. Situations call upon us to be courageous, to be fair, to be compassionate, and each of these virtues requires the situationally appropriate expression of some typical human capacity for pursuing the good. As we have discussed, to be courageous is to take appropriate risks for the sake of a worthwhile end; to be fair is to give others their due in situations in which inappropriate favoritism is possible; and to be compassionate is to exercise sensitivity to the concerns of others in situations involving suffering. The excellence that marks out virtuous action from ordinary actions (Broadie, 1991) partly consists in the ways that practical wisdom helps us to recognize which virtue or virtues are called for, to integrate and prioritize multiple virtues, to decide what will make an action virtuous according to one’s best understanding of a good life (blueprint), and to allow one’s emotional experience and behavior to be guided by reason. A person lacking one or more components of practical wisdom might not be able to detect that courage and compassion are called for, or they would be unable to determine the right combination of risk-taking or perspective-taking required by the given situation, or they would be unable to recognize how courage and compassion fit into a life plan, or they might get carried away by emotion and miss the opportunity for courageous and compassionate action. In sum, a person without practical wisdom cannot be consistently virtuous. This illustrates practical wisdom’s important role as a centerpiece in any virtue-theoretic interpretation of human activities and illuminates the pressing relevance of the concept for our troubled times.

## Practical Wisdom as a Pathway to Flourishing Through Frailty

Even in ordinary times, human life is very complex. The advent of a very contagious virus that can be deadly has amplified that complexity significantly and introduced a host of new questions and issues about personal and public safety. Humans are relatively easy to overwhelm and tend to act following fast, intuitive judgments that involve many biases and shortcuts (Kahneman, 2011). In addition, strong emotions such as fear and disgust can overwhelm more reasoned approaches to problems, and these emotions can be primary sources of confusion and impulsivity. When we add the multiplicity of human goods (e.g., friendship, pleasure, justice, social status, social harmony, etc.) and the multiple individual and group perspectives and interests to this complexity, it seems miraculous that human beings can act on a coherent plan at all. We have focused on reasoned choices as a key element of virtue and therefore of flourishing. There is, however, substantial evidence that a great many human actions are based on quick, automatic cognitive and emotional processes that do not involve the conscious, deliberate thought usually associated with reasoned action. Given the complexity and pace of human life, these automatic processes seem necessary, for if one needed to engage conscious deliberation for every decision, life would come to a grinding halt. Yet quick, automatic actions often create difficulties because they can lead to interpersonal or intergroup conflict or undermine longer-term plans and activities. Therefore, the complexity of life reveals many human frailties.

Practical wisdom is the capacity to deal excellently with complexity. Aristotle (1999) gave the following definition: “practical wisdom is a truthful rational characteristic of acting in matters involving what is good for man [*sic*]” (1140b 20–21). That seems like a very tall order, but if we look beyond Aristotle’s somewhat perfectionistic tone, we can see how practical wisdom can help. He said that we must be honest and rational as we assess our circumstances to determine how to act. By focusing on the key word “rational” we can begin to understand what that means for ordinary humans. By “rational,” Aristotle did not mean acting completely logically or systematically. Rather, he meant that people act according to reasons, that decisions and actions are best undertaken with good reasons. And he clarified that the best reasons for action are the human ends or goods that make life worthwhile, suggesting the incumbency of acting for the sake of human goods such as friendship, justice, and safety. Many of our actions will generally remain automatic and intuitive, such as activities related to driving to meet a friend or locking a door after entering the house or leaving a car. Therefore, automatic actions serve the more important ends. Moreover, the goal of virtue development is to make virtuous actions (e.g., kindness and fairness) automatic, to make them second nature. The key thing is to recognize that there is no inherent opposition or eliminative relation between quick, automatic actions and more deliberative ones. Each has its place, and they can be seen much more sensibly as complementary rather than taking an “either/or” view.

Practical wisdom helps with complexity by focusing our attention on the most central aspects of our circumstances so that we can act on the most important matters rather than being distracted by more trivial or eye-catching elements. The moral perception function of practical wisdom helps us identify what sort of situation we are facing, which suggests one or more relevant virtues. That makes it possible to organize, synthesize, or prioritize the relevant virtues, if needed. The actor’s overall understanding of what is worthwhile in life further motivates and guides action, and the reasons for acting one way rather than another help one to shape and direct our emotions in the best ways. Practical wisdom allows us to address a great deal of complexity by focusing our attention on the most important elements of our situation in view of our best understanding of how action in that situation can contribute to a good life. To see the importance of practical wisdom, consider the mayhem created by foolish decisions and short-sightedness.

A salient contemporary example of the value of practical wisdom can be found in current disputes about policing. This contentious domain includes demands that “Black lives matter,” which are met with counter-chants that “blue lives matter” and “defund the police” vs. “support the police.” These questions have typically been framed in very partisan ways and often in either/or terms. From a practical wisdom perspective, any temptation to enter such an “either/or” resolution should immediately be suspect because a key function of practical wisdom is to harmonize multiple concerns rather than plunking for one simple solution or the other.

The point that Black lives matter activists are making is that, in everyday policing practices, Black people’s lives are clearly far more often disrupted or ended than White lives. The point that blue lives matter voices make is that police frequently risk their safety and even lives in the service of their communities. Both points seem reasonable, true, and worthy of honoring. The blueprint function of practical wisdom can then direct our attention to what the valued aim of policing is. Two candidates for the aim of policing are law enforcement or the maintenance of social order. Maintaining social order is certainly part of policing, but that could all too easily evoke the specter of tyranny or white supremacy as the order being maintained. Indeed, the disproportionate police violence toward people of color seems to many as an expression of a racial hierarchy. Law enforcement is a common understanding of the aim of policing, but, as we see it, it is a means, not an end.

The ends of law enforcement, on our view, are public safety and societal justice^2^. Once we take seriously the idea that all lives matter, public safety acquires new meaning. It is safety for the *entire* public, and violence toward one member of society means that all members of society are at risk. Law enforcement is also a means toward the constitutive end of societal justice. It is a constitutive end because justice is both the desired aim and the necessary means. One can only enact justice through just actions, and social justice demands that everyone in society be treated justly. Equality before the law is a bedrock principle for democracies everywhere, and when “law enforcement” results in discriminatory practices toward a subset of society, the means has gone awry and is no longer serving societal justice. These are difficult times as we grapple anew with centuries of oppression and maltreatment. The best way forward is to keep our treasured ideals of public safety and justice foremost in our minds and to allow that blueprint to guide our attention to the aspects of our circumstances that will promote just actions and harmonize those actions with everyone’s safety.

## Conclusion

COVID-19 has been enormously disruptive and has created severe strains on individual, relational, and societal well-being. It has strained the world economy and wreaked havoc on many people’s basic abilities to make a living and maintain a home. Although life is seldom easy, it has been especially difficult for the past year. Fowers et al. (2017) argued that humans have multiple frailties, and that the virtues are the characteristics that make it possible to flourish through those frailties. We have applied that viewpoint to three rampant and salient aspects of the pandemic: risk, injustice, and complexity. The virtue associated with fear and risk is courage, which is the appropriate degree of risk-taking, given the valued ends at stake. In this pandemic, lives and health are at risk, so extraordinary measures have been necessary. One of the key actions that has prolonged the pandemic is the refusal to recognize the risks and act with appropriate caution and safety protocols. Notably, the simple precaution of wearing face masks has been widely flouted in the United States, and this has been encouraged by several prominent political and social figures. The pandemic has also accentuated the recognition of injustices in our societies and protests against those injustices. The virtue of justice is called for to increase the kind of just actions to rectify historic imbalances that have continued into the present.

Practical wisdom, a meta-virtue that guides the expression of other virtues, is the trait that can help us to address confusion and complexity with excellence. One of the things that has made COVID-19 difficult is that there are many valued ends that have been put at risk by the pandemic, including, of course, health, but also livelihoods, belonging, education, and mental health. For example, it has proven extremely difficult for the government and people of the United States to balance these goods, with some prospering tremendously during the pandemic and many others losing their businesses, incomes, and even their dwellings. The inability to balance valued ends has cost our society and many individuals dearly, and we can only hope that practically wise leadership and followership will increase.

We chose to focus on courage, justice, and practical wisdom because they are so salient in the pandemic and so that we could give a reasonably in-depth account. There are many other virtues that have come to the fore in 2020 as well. For example, the ramping up of free food distribution and the many extraordinary kindnesses that have been observed also emphasize the virtues of generosity and compassion. On reflection, this pandemic, like virtually all other human struggles, can be characterized in Martin Luther King’s (King, n.d.) immortal words: “Every crisis has both its dangers and its opportunities. Each can spell either salvation or doom”. To the degree that we practice the virtues that conduce to public health and other goods, we maintain or increase the likelihood of flourishing. To the degree that we allow ourselves to practice deficiencies or excesses (e.g., cowardice or recklessness) relative to virtues, we increase our tendency to languish. Each of us makes many choices everyday that can guide us as individuals and as a society to flourish or to languish, so opportunities for virtuous actions and the pursuit of worthwhile ends abound, even, or especially, in a pandemic.

## Author Contributions

BF conceptualized the manuscript, oversaw the contributions of the other authors, independently wrote 25% of first draft of the manuscript, and edited the penultimate and ultimate versions of the manuscript. LN wrote 25% of the first draft of the manuscript. AC wrote 25% of the first draft of the manuscript. RS wrote 25% of the first draft of the manuscript. All authors contributed to the article and approved the submitted version.

## Conflict of Interest

The authors declare that the research was conducted in the absence of any commercial or financial relationships that could be construed as a potential conflict of interest.

## Footnotes

1 Unfortunately, the term *justice* is commonly used to refer to many things. For our purposes, we will refer to justice in two ways, following Aristotle’s (1999) usage. First, as a set of societal arrangements that can be evaluated based on the equity and equality they represent. That is the focus of this section of the paper. Second, we will refer to justice as a virtue, which we describe as an acquired trait that is properly motivated and enacted by an individual (see the next major section of the paper.). We endeavor to keep these two usages distinct.

2 Recall that in a neo-Aristotelian framework, the term *justice* can refer to both a virtue that one can cultivate and to the aim of a fair social arrangement.

## References

[B1] Aristotle (1999). *Nicomachean Ethics* (M. Ostwald, trans.). Upper Saddle River, NJ: Prentice Hall. 10.1093/oseo/instance.00258595

[B2] Broadie S. (1991). *Ethics with Aristotle.* Oxford: Oxford University Press.

[B3] Centers for Disease Control and Prevention (2020). *CDC COVID Data Tracker.* Atlanta, GA: Centers for Disease Control and Prevention.

[B4] Cokelet B. Fowers B. J. (2019). Realistic virtues and how to study them: introducing the STRIVE-4 model. *J. Moral Educ.* 48 7–26. 10.1080/03057240.2018.1528971

[B5] Darnell C. Gulliford L. Kristjánsson K. Paris P. (2019). Phronesis and the knowledge-action gap in moral psychology and moral education: a new synthesis? *Hum. Dev.* 62 101–129. 10.1159/000496136

[B6] Di Martino S. Prilleltensky I. (2020). Happiness as fairness: the relationship between national life satisfaction and social justice in EU countries. *J. Commun. Psychol.* 48 1997–2012. 10.1002/jcop.22398 32627203

[B7] Dunne J. (1993). *Back to the Rough Ground: ‘*Phronesis’ *and* ‘Techne’ *in Modern Philosophy and in Aristotle.* Notre Dame, IN: University of Notre Dame Press.

[B8] Fowers A. F. Wan W. (2020). *A Third of Americans Now Show Signs Of Clinical Anxiety Or Depression, Census Bureau Finds Amid Coronavirus Pandemic*. Washington, DC: The Washington Post.

[B9] Fowers B. J. (2008). From continence to virtue: recovering goodness, character unity, and character types for positive psychology. *Theory Psychol.* 18 629–653. 10.1177/0959354308093399

[B10] Fowers B. J. (2015). *The Evolution of Ethics: human Sociality and the Emergence of Ethical Mindedness.* London: Palgrave/McMillan.

[B11] Fowers B. J. (2017). “The deep psychology of eudaimonia and virtue: belonging, loyalty, and the Anterior Cingulate Cortex,” in *Varieties of Virtue*, eds Carr D. Arthur J. Kristjánsson K. (London: Palgrave/MacMillan), 199–216. 10.1057/978-1-137-59177-7_12

[B12] Fowers B. J. Carroll J. S. Leonhardt N. D. Cokelet B. (2021). The emerging science of virtue. *Perspect. Psychol. Sci.* 16 118–147. 10.1177/1745691620924473 32835627

[B13] Fowers B. J. Richardson F. C. Slife B. D. (2017). *Frailty, Suffering and Vice: Human Flourishing in the Face of Limitations.* Washington, DC: APA Books. 10.1037/0000035-000

[B14] Gale M. M. Pieterse A. L. Lee D. L. Huynh K. Powell S. Kirkinis K. (2020). A meta-analysis of the relationship between internalized racial oppression and health-related outcomes. *Couns. Psychol.* 48 498–525. 10.1177/0011000020904454

[B15] Gallagher M. W. Smith L. J. Richardson A. L. D’Souza J. M. Long L. J. (2021). Examining the longitudinal effects and potential mechanisms of hope on covid-19 stress, anxiety, and well-being. *Cogn. Behav. Ther.* 10.1080/16506073.2021.1877341 [Epub ahead of print]. 33544032

[B16] Grossmann I. Weststrate N. M. Ardelt M. Brienza J. P. Dong M. Ferrari M. (2020). The science of wisdom in a polarized world: knowns and unknowns. *Psychol. Inq.* 31 103–133. 10.1080/1047840X.2020.1750917

[B17] Henrich N. Henrich J. (2007). *Why Humans Cooperate: A Cultural and Evolutionary Explanation.* Oxford: Oxford University Press.

[B18] Jankowski P. J. Sandage S. J. Bell C. A. Davis D. E. Porter E. Jessen M. (2020). Virtue, flourishing, and positive psychology in psychotherapy: an overview and research prospectus. *Psychotherapy* 57 291–309. 10.1037/pst0000285 31985238

[B19] Johns Hopkins University Coronavirus Resource Center (2020). *COVID-19 Dashboard by the Center for Systems Science and Engineering at Johns Hopkins University.* Available online at: https://coronavirus.jhu.edu/map.html (accessed December 21, 2020).

[B20] Kahneman D. (2011). *Thinking, Fast and Slow.* New York, NY: Farrar, Straus and Giroux.

[B21] Kinder M. (2020). *Meet the COVID-19 Frontline Heroes.* Washington, DC: Brookings.

[B22] King M. L. Jr. (n.d.). “The quest for peace and justice,” in *Nobel Lecture*, https://www.nobelprize.org/prizes/peace/1964/king/lecture/

[B23] Krebs C. C. (2020). *Memorandum on Identification of Essential Critical Infrastructure Workers During COVID-19 Response.* Rosslyn, VA: U. S. Department of Homeland Security, Cybersecurity & Infrastructure Security Agency.

[B24] Kristjánsson K. Darnell C. Pollard D. Fowers B. (2021). Phronesis as a type of contextual integrative thinking: a unique source of wise moral motivation or a redundant construct? Manuscript in review.

[B25] LeDoux J. E. (2012). Evolution of human emotion: a view through fear. *Prog. Brain Res.* 195 431–442. 10.1016/B978-0-444-53860-4.00021-0 22230640 PMC3600914

[B26] Long H. Van Dam A. Fowers A. Shapiro L. (2020). *The Covid-19 Recession is the Most Unequal in Modern U.S. History.* Washington, DC: The Washington Post.

[B27] MacIntyre A. C. (1988). *Whose Justice? Which Rationality?* Notre Dame, IN: University of Notre Dame Press.

[B28] Munasinghe S. Sperandei S. Freebairn L. Conroy E. Jani H. Marjanovic S. (2020). The impact of physical distancing policies during the COVID-19 pandemic on health and well-being among Australian adolescents. *J. Adolesc. Health* 67 653–661. 10.1016/j.jadohealth.2020.08.008 33099413 PMC7577185

[B29] Newstead T. Macklin R. Dawkins S. Martin A. (2018). What is virtue? Advancing the conceptualization of virtue to inform positive organizational inquiry. *Acad. Manag. Perspect.* 32 443–457. 10.5465/amp.2016.0162

[B30] Ngamaba K. H. Panagioti M. Armitage C. J. (2018). Income inequality and subjective well-being: a systematic review and meta-analysis. *Qual. Life Res.* 27 577–596. 10.1007/s11136-017-1719-x 29067589 PMC5845600

[B31] Peterson C. Seligman M. E. P. (2004). *Character Strengths and Virtues: A Handbook and Classification.* Oxford: Oxford University Press.

[B32] Stichter M. (2018). *The Skillfulness of Virtue.* Cambridge: Cambridge University Press. 10.1017/9781108691970

[B33] United States Census Bureau (2020). *Race.* Suitland, MD: United States Census Bureau.

[B34] West S. A. Griffin A. S. Gardner A. (2007). Evolutionary explanations for cooperation. *Curr. Biol.* 17 R661–R672. 10.1016/j.cub.2007.06.004 17714660

[B35] WHO (2021). *WHO Coronavirus Disease (COVID-19) Dashboard.* Available online at: https://covid19.who.int/ (accessed February 18, 2021).

[B36] Wilkinson R. Pickett K. (2020). *The Inner Level: How More Equal Societies Reduce Stress, Restore Sanity and Improve Everyone’s Well-Being.* London: Penguin press. 10.3399/bjgp19X705377 PMC6715479 31467020

[B37] Wong P. T. (2011). Positive psychology 2.0. *Can. Psychol.* 52 69–81. 10.1037/a0022511

[B38] Wong P. T. (2017). Meaning-centered approach to research and therapy, second wave positive psychology, and the future of humanistic psychology. *Humanist. Psychol.* 45 207–216. 10.1037/hum0000062

[B39] Yıldırım M. Akgül O. Geçer E. (2021). The effect of COVID-19 anxiety on general health: the role of COVID-19 coping. *Int. J. Mental Health Addict.* 10.1007/s11469-020-00429-3 [Epub ahead of print]. 33456406 PMC7799156

